# AlloBench: A Data Set Pipeline for the Development and Benchmarking of Allosteric Site Prediction Tools

**DOI:** 10.1021/acsomega.5c01263

**Published:** 2025-04-23

**Authors:** Dibyajyoti Maity, Baofu Qiao

**Affiliations:** Department of Natural Sciences, Baruch College, City University of New York, New York 10010, New York United States

## Abstract

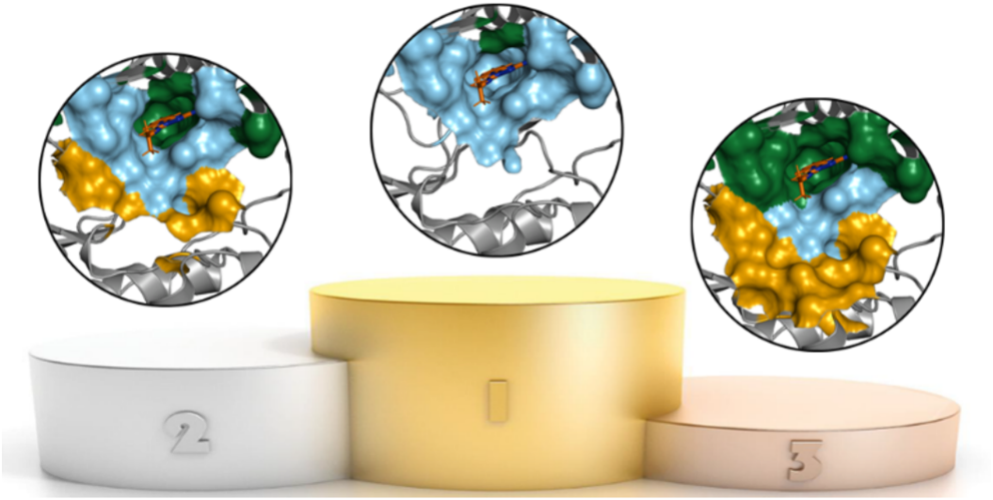

Allostery refers to the activity regulation of biological macromolecules originating from the binding of an effector molecule at the allosteric site that is distant from the active site. The few existing allosteric data sets have not been updated with recent discoveries of allosteric proteins and are challenging to use for data-intensive tasks. Instead of providing another data set bound to become outdated, we present the AlloBench pipeline to create high-quality data sets of biomolecules with allosteric and active site information suitable for computational and data-driven studies of protein allostery. The pipeline produces a data set of 2141 allosteric sites from 2034 protein structures with 418 unique protein chains by integrating information from AlloSteric Database, UniProt, Mechanism and Catalytic Site Atlas, and Protein Data Bank. Furthermore, we use a subset of 100 proteins from the AlloBench data set to quantitatively compare the performance of currently available allosteric site prediction tools: APOP, PASSer, Ohm, ALLO, Allosite, STRESS, and AlloPred. Such a large-scale benchmarking of these programs has not been undertaken on a common test set. The results show a significant need for improvement, as the accuracy for all programs is well below 60%, with PASSer (Ensemble) outperforming the rest. The AlloBench pipeline will not only promote the development of improved allosteric site prediction tools but also serve as a reference for studying allostery in general.

## Introduction

Allostery is the activity regulation of biological macromolecules originating from the binding of an effector molecule at the allosteric site that is distant from the active site, as opposed to the active site where the substrate binds.^1^ Identifying allosteric sites is essential for a fundamental understanding of biomolecular signaling and enzymatic activity.^2−6^ Also, drug molecules targeting the allosteric sites offer significant advantages compared to orthosteric drugs, such as excellent selectivity, tuneability, and fewer side effects, as they do not compete with the substrate and are not subject to the same evolutionary conservation pressures as the active site.^7^ In addition, allosteric modulators can both upregulate and downregulate the activity of the target protein.^5,6,8^

Allosteric sites and interactions have been found in many proteins by separate studies, each on one or a few proteins. The AlloSteric Database (ASD) was the first to collate such information into a comprehensive database.^9^ The latest version of ASD (2023) provides 3102 allosteric sites, 748 allosteric protein–protein interaction modulators that target 50 specific interactions, a data set of 1312 allosteric mutations, an “Allosteric Hit-to-Lead” data set with 91 small molecule hits and 6851 leads, 490 dualsteric modulators for simultaneous interaction with allosteric and orthosteric sites, AlloSite-Potential (the data set of potential allosteric sites in 17,627 human proteins predicted using AlloSitePro), the human allosteric pocketome with 261 allosteric networks, 538 allosteric drugs in Allo-Drug data set, and 57 allosteric pathways.^10^ ASD was compiled from the scientific literature by filtering the PubMed abstracts with the keyword “alloster*”, which would match mentions of allostery and allosteric. The names of the allosteric proteins extracted from the abstracts were clustered. The obtained literature was manually reviewed for at least three pieces of evidence of allosteric regulation from biochemical experiments, including protein structures bound to the allosteric modulator.^9^ Recently, ASD has added a subset with allosteric and orthosteric sites to their website at https://mdl.shsmu.edu.cn/ASD/module/site/site.jsp, but the downloadable files from ASD 2023 do not contain the orthosteric site residues. Moreover, only 46% of entries have annotations for orthosteric sites, and only 209 unique orthosteric sites are present. Each orthosteric site is available for download only in the Protein Data Bank (PDB) format. The PDB file contains the residues for the site, which must be processed and synchronized with the target protein. In comparison, AlloBench adds active site annotation to 65% of the ASD entries and 446 out of the 699 unique protein chains in ASD 2023 (UniProt IDs).

ASBench and CASBench were later derived from ASD to remove the redundancy and quality issues in ASD. ASBench, released by the same group that created ASD,^11^ contains two benchmarking data sets: the “Core set” with 235 unique allosteric sites in 202 unique proteins and the “Core-Diversity set” with 147 structurally diverse allosteric sites in 127 unique proteins. First, the proteins from ASD 2014 with structures having a resolution better than 3.0 Å were selected. Structures with covalently bound allosteric modulators were removed. Structures were also removed if the allosteric modulators were peptides or ions, had overlapping allosteric and active sites, or had missing residues within 4 Å of the allosteric modulator. The “Core set” was obtained by selecting the structure with the most interactions between the protein and allosteric modulator, evaluated using Ligplot+^12^ for each allosteric site, and choosing the best resolution structure in case of ties. The “Core-Diversity set” was obtained by clustering the allosteric sites using the pocket similarity score (PS-Score) from the pairwise structural alignment of the allosteric sites using APoc.^13^ The best resolution protein structure from each cluster obtained at a cutoff of 0.5 PS-Score was selected to represent the cluster. Note that ASBench only provides allosteric sites and does not include the active site information.

On the other hand, CASBench contains 2870 protein data bank (PDB) structures from 91 unique proteins and thus has significant redundancy.^14^ First, the allosteric site residue numbers from ASD were synchronized with the corresponding PDB structure. Then, all available structures of the ASD proteins from the PDB were obtained, and their sequences were clustered using the CD-HIT program at 95% sequence similarity. Only those clusters were retained where at least one of the proteins was present in the Catalytic Site Atlas (CSA). Finally, the allosteric and catalytic site annotations were obtained by identifying the ligands bound within 5 Å of the allosteric or catalytic site residues and then detecting all residues within 5 Å of the ligand to determine the pocket. The essential differences between ASBench, CASBench, and the present work are compiled in Table S1.

**Figure 1:**
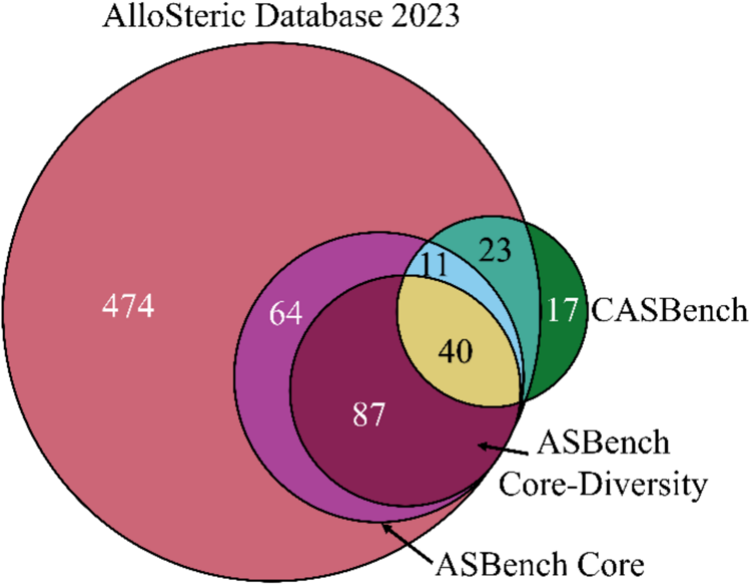
Venn diagram of unique protein chains (UniProt accession number) in each data set. The numbers are reported for each intersection. The AlloSteric Database 2023 contains 699 proteins. The Core and Core-Diversity data sets of ASBench contain 202 and 127 proteins, respectively. CASBench is comprised of 91 proteins.

ASBench and CASBench have become outdated and are significantly smaller compared to the latest version of ASD 2023 (Figure 1). Due to the lack of updated data sets, new programs (especially AI-based tools) being developed for studying allostery are still using the outdated ASBench data set released 10 years ago (Table 1). Although ASBench lists the allosteric site residues on its web server, such information is missing in the downloadable data set but needs to be extracted from the structure files. Furthermore, ASBench does not contain the active site residues of the target proteins, which were resolved in CASBench by adding the active site residues from the CSA (superseded by M-CSA). However, only 57 unique protein chains from ASD are present in the Mechanism and Catalytic Site Atlas (M-CSA), while the current version of UniProt has active site residues for 629 out of 690 unique protein chains in the ASD, including 54 of 57 proteins common between M-CSA and ASD. While CASBench does list multiple active sites and allosteric sites, the corresponding ligands or allosteric modulators are absent, which must be inferred from the PDB structure.

**Table 1:** Summary of the Allosteric Site Prediction Tools

program	year	pocket detection	method	training data	web server	source code	refs
APOP	2023	Fpocket	normal mode analysis		https://apop.bb.iastate.edu	https://github.com/Ambuj-UF/APOP	(15)
PASSer (Rank)	2023	Fpocket	learning-to-rank	Allosite data set +138 proteins from ASBench Core-Diversity set	https://passer.smu.edu		(16)
PASSer (AutoML)	2022	Fpocket	AutoML with AutoGluon	Allosite data set +138 proteins from ASBench Core-Diversity set	https://passer.smu.edu	https://github.com/smu-tao-group/PASSer2.0	(17,18)
PASSer (Ensemble)	2021	Fpocket	ensemble of XGBoost + graph convolution network	90 proteins from ASD (same as the Allosite data set)	https://passer.smu.edu		(19)
Ohm	2019		perturbation analysis		https://dokhlab.med.psu.edu/ohm/#/	https://bitbucket.org/dokhlab/ohm/src/master	(20)
ALLO	2018	DoGSiteScorer 2.0	naïve Bayes classifier + artificial neural network	95 proteins from the ASBench Core-Diversity set		http://github.com/fibonaccirabbits/allo	(21)
AllositePro	2016	Fpocket	normal mode analysis + logistic regression	ASBench Core-Diversity set (147 proteins)	https://mdl.shsmu.edu.cn/AST		(22)
STRESS	2016	Monte Carlo search using pseudo ligand	normal mode analysis			https://github.com/gersteinlab/STRESS	(23)
AlloPred	2015	Fpocket	normal mode analysis + support vector machine	140 proteins from the ASBench Core-Diversity set		https://github.com/jgreener64/allopred	(24)
Allosite	2013	Fpocket	support vector machine	90 proteins from ASD	https://mdl.shsmu.edu.cn/AST		(25)

**Figure 2:**
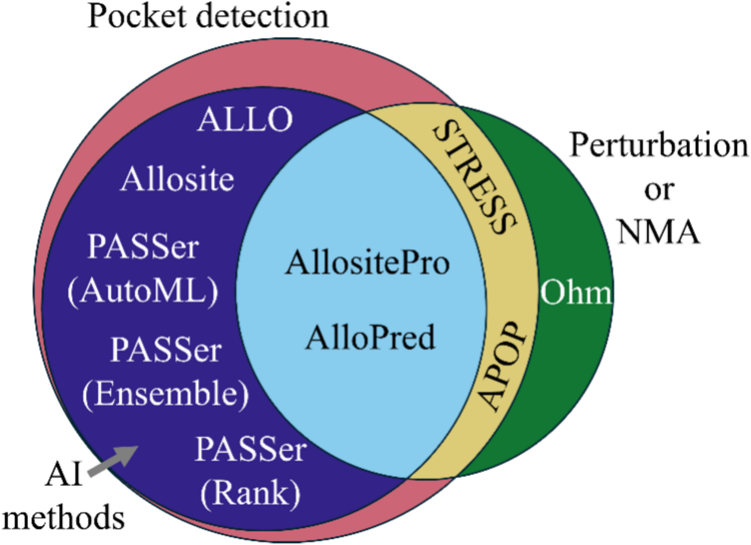
Venn diagram illustrating the various methods used in each allosteric site prediction tool. Pocket-based methods first identify the pockets on the protein surface and then evaluate whether the pockets are allosteric sites. AI methods use machine learning models trained on pocket features. The perturbation methods primarily rely on normal-mode analysis to either obtain features to pass to the ML model or directly evaluate the effect of perturbing the pocket on protein dynamics.

The availability of these data sets sparked the development of numerous computational approaches to predicting the allosteric site in proteins. The allosteric site prediction tools studied here have been summarized in Table 1, and their details are provided in the Supporting Information. The AI methods (Figure 2) first use a pocket detection tool to identify the pockets on the protein surface and then use machine learning (ML) models trained on the features obtained from the pocket detection to evaluate whether each candidate pocket may be an allosteric site. In contrast, the perturbation methods APOP, Ohm, and STRESS assess the change in dynamics of the protein from the simulated binding of the allosteric modulator to rank the candidate pockets. APOP and STRESS use normal-mode analysis (NMA), while Ohm uses a stochastic algorithm to calculate the perturbations. AllositePro and AlloPred use features from NMA in their ML models in addition to pocket features to incorporate the perturbation propagation information. Note that Ohm and AlloPred need the active site residues to run, while others do not. Hence, having both the active site and allosteric site residues is critical.

The accuracy of these tools was primarily evaluated independently in their respective publications. Allosite (and AllositePro) reported the performance metrics like sensitivity, specificity, and precision of the underlying scoring function or the artificial intelligence (AI) model that classifies a pocket on the protein surface as allosteric or nonallosteric.^25^ In contrast, AlloPred and APOP reported the number or percentage of cases in which the known allosteric pocket appeared to be at the topmost or among the top three predictions. PASSer (all three models of Ensemble, AutoML, and Rank) and ALLO reported the results from both approaches. Previous comparisons evaluated the performance of a few programs on a few protein cases.^5,26−29^ However, a systematic study quantitatively comparing the performance of the available allosteric site prediction tools on a common test set has yet to be performed. Previously, the pocket detection and binding site prediction tools ConCavity,^30^ DeepSite,^31^ DoGSite3,^32^ and SAPocket^33^ used the Jaccard index (JI) of the volumes of the known and predicted sites to evaluate the accuracy. However, JI has not been adopted to report the accuracy of allosteric site prediction tools.

To address the lack of high-quality data on allosteric proteins, we present the AlloBench data set pipeline. This pipeline uses ASD, UniProt, and PDB to create a set of proteins with known allosteric and active site residues. AlloBench fixes the critical issues in ASD 2023 by automated checking of allosteric and active site annotations, updating the obsolete PDB and UniProt IDs, filtering for high-resolution protein structures, and other checks as described in the “AlloBench Pipeline” Section of the methods. The primary benefit of having a pipeline instead of a static data set is that it can be updated with further checks in the future as and when more issues are identified. The tabular output from the AlloBench pipeline in CSV format can be easily filtered either by modifying the Jupyter notebook or using programs like Microsoft Excel to create unbiased subsets depending on the use case. Also, the gaps in the target proteins were reconstructed using the ProMod3^34^ modeling engine to make them suitable for computational and dynamics-based studies. Furthermore, we demonstrate the utility of AlloBench by benchmarking the available allosteric site prediction tools: PASSer, ALLO, AlloPred, Allosite, AllositePro, APOP, Ohm, and STRESS on a subset of 100 proteins. We show that the JI of the known and predicted allosteric site residues is a simple yet powerful metric to quantitatively evaluate the accuracy of the various allosteric site prediction programs and web servers. PASSer (Ensemble) proved to be the best among the programs studied here, but they all need improvement.

## Methods

### AlloBench Pipeline

**Figure 3:**
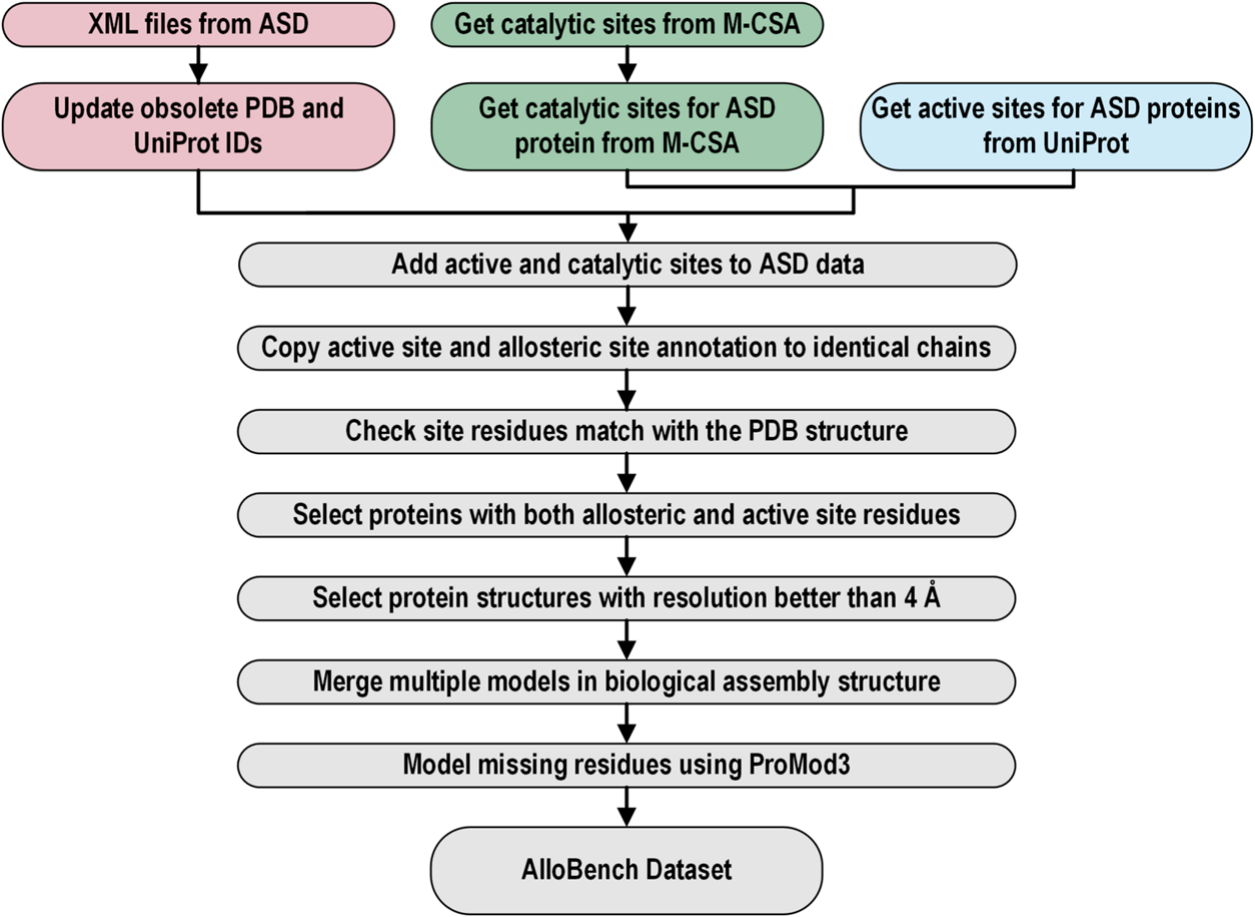
Schematic illustrating the steps in the AlloBench pipeline.

The steps of the AlloBench pipeline are illustrated in Figure 3. Proteins with known allosteric sites were obtained from the ASD and are available as XML files or a table in a tab-delimited text file. Unfortunately, the tab-delimited file had missing values in the column for allosteric site residues for 1620 out of 3102 entries, so the XML files were downloaded and parsed to extract the data. Obsolete PDB IDs of the target protein were updated, and ASD entries of structures no longer present in the PDB were dropped. Next, UniProt IDs were updated by fetching the data using PDB’s GraphQL API, and the entries with discrepancies between PDB and UniProt IDs were removed.

ASD does not provide the active site residues necessary to run AlloPred and Ohm. Thus, these were obtained from the UniProt and M-CSA^35^ databases and merged. The active site residue numbers are from the UniProt sequence. Although these match the residue numbering in the PDB structure for most proteins, there were discrepancies in some instances, especially for modeled structures. This was resolved by aligning the UniProt sequence with the PDB sequence and identifying the correct residue number in the PDB structure. The entries containing both the allosteric and active site residues that match the residue numbering of the PDB structure were selected and further filtered to only include PDB structures with a resolution better than 4 Å.

The biological assembly structures of the target proteins were downloaded from the PDB website (www.rcsb.org). A few proteins available only in mmCIF format were removed, as the allosteric site prediction tools do not support structure file formats other than PDB. 613 structures contained multiple models in their PDB files. The models in the respective PDB files were merged into a single model while ensuring the unique labeling of chains. 1291 out of 2034 structures had missing residues, and 1287 structures could be modeled using ProMod3, the modeling engine behind the SWISS-MODEL web server.^36,37^ ProMod3 was chosen because it can model multimeric proteins if the target-template alignment is provided in a JSON file. The PDB structure was used as the template, and the sequence from the SEQRES records in the PDB file was the target sequence. The reliability of the models was evaluated by calculating the lDDT (Local Distance Difference Test)^38^ between the modeled structure and the original PDB structure. Modeled structures with lDDT < 0.8 were removed from the data set. The discrepancies between the allosteric sites listed in ASD and the location of the allosteric modulator in the PDB structure were resolved by obtaining the chemical component alias of the allosteric modulators in the protein structure from the ASD. Then, allosteric sites were obtained by locating the residues within 4 Å of the allosteric modulator in the PDB structures.

### Test Set Preparation

The proteins in the training sets of the AI-based allosteric site prediction tools were identified as described in Supporting Information Section S1. This also included protein from ASBench and CASBench, which were used to train many of the allosteric site prediction tools in Table 1. The UniRef50 cluster IDs (UniProt reference clusters with at least 50% sequence identity) were obtained for the 268 unique UniProt IDs of these proteins. AlloBench proteins with these UniRef50 cluster IDs were dropped to remove any related proteins in addition to the proteins of the training sets. Only the entries with small-molecule allosteric modulators were selected since the tools evaluated here have been primarily designed to predict allosteric sites occupied by small molecules. Additionally, enormous structures with more than 8000 residues (e.g., 5U03, 5U6R, 5U3C) and structures containing nucleic acids (e.g., 5NPP) were dropped. Finally, the heteroatoms in the PDB files were removed, and only the protein chains were retained to create a test set of 100 protein structures.

### Running Allosteric Site Prediction Tools

The allosteric site prediction tools were run for each protein in the test set using the procedure detailed in Section S2 of the Supporting Information. PASSer (Rank, AutoML, and Ensemble) was run by automating the submission using its API. APOP and Ohm were installed and run locally from the source code to automate the process instead of manually submitting each protein to the web servers. Allosite and AllositePro do not provide any source code or local executables, so each protein had to be manually submitted to the web server, and the prediction reports were downloaded. ALLO, STRESS, and AlloPred do not have a web server, so these were installed and run locally following the instructions in the Supporting Information.

APOP, ALLO, Ohm, and PASSer produced output for all 100 structures in the test set. However, the three PASSer models (Ensemble, AutoML, and Rank) returned spurious residue numbers alongside proper residue numbers in at least one of their top 3 allosteric site predictions for 13, 14, and 13 proteins, respectively (see Supporting Information Table S3). The spurious residues were removed from the predicted allosteric site residues while retaining the correct ones. AllositePro, STRESS, AlloPred, and Allosite failed for 8, 9, 6, and 4 proteins in the test set, respectively (see Supporting Information Table S3).

### Quantitative Comparison of Predicted Allosteric Sites

The output from all allosteric site prediction tools is a list of residues of the pocket for the allosteric site. The agreement between the known and predicted allosteric sites was assessed using the Jaccard index (JI),^39,40^ which is given by


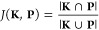
where **K** and **P** are the sets of residues in the known and predicted allosteric sites, respectively. The JI can have a value between 0 and 1. It is 0 if there are no residues in common between K and P and is 1 if K and P are identical. Also, the JI is low if the predicted set has too many residues that are not a part of the known set. Only the topmost prediction from each program was considered to keep the comparison uniform, as Allosite, AllositePro, and STRESS returned less than three predictions for some proteins. The probabilities reported by ALLO, Allosite, PASSer (AutoML), and PASSer (Ensemble) or the scores by AllositePro, APOP, and PASSer (Rank) were also not utilized here for the purpose of comprehensive comparison.

We also compared the JI of the predicted allosteric site to the inverse of the distance between the centroids of the Cα atoms of residues in the predicted allosteric sites to the known allosteric sites to estimate the proximity of the two sites in three-dimensional (3D) space (Figure S1). In the case of multimeric proteins with multiple known allosteric sites, the topmost prediction from an allosteric site prediction tool was compared to all the known allosteric sites, and the one with the highest JI was used. The strong correlation between the JI and inverse distance indicates that the JI is a robust estimator of the proximity of the known and predicted allosteric site, in addition to measuring the overlap between the sites.

## Results

### AlloBench Data Set

**Figure 4:**
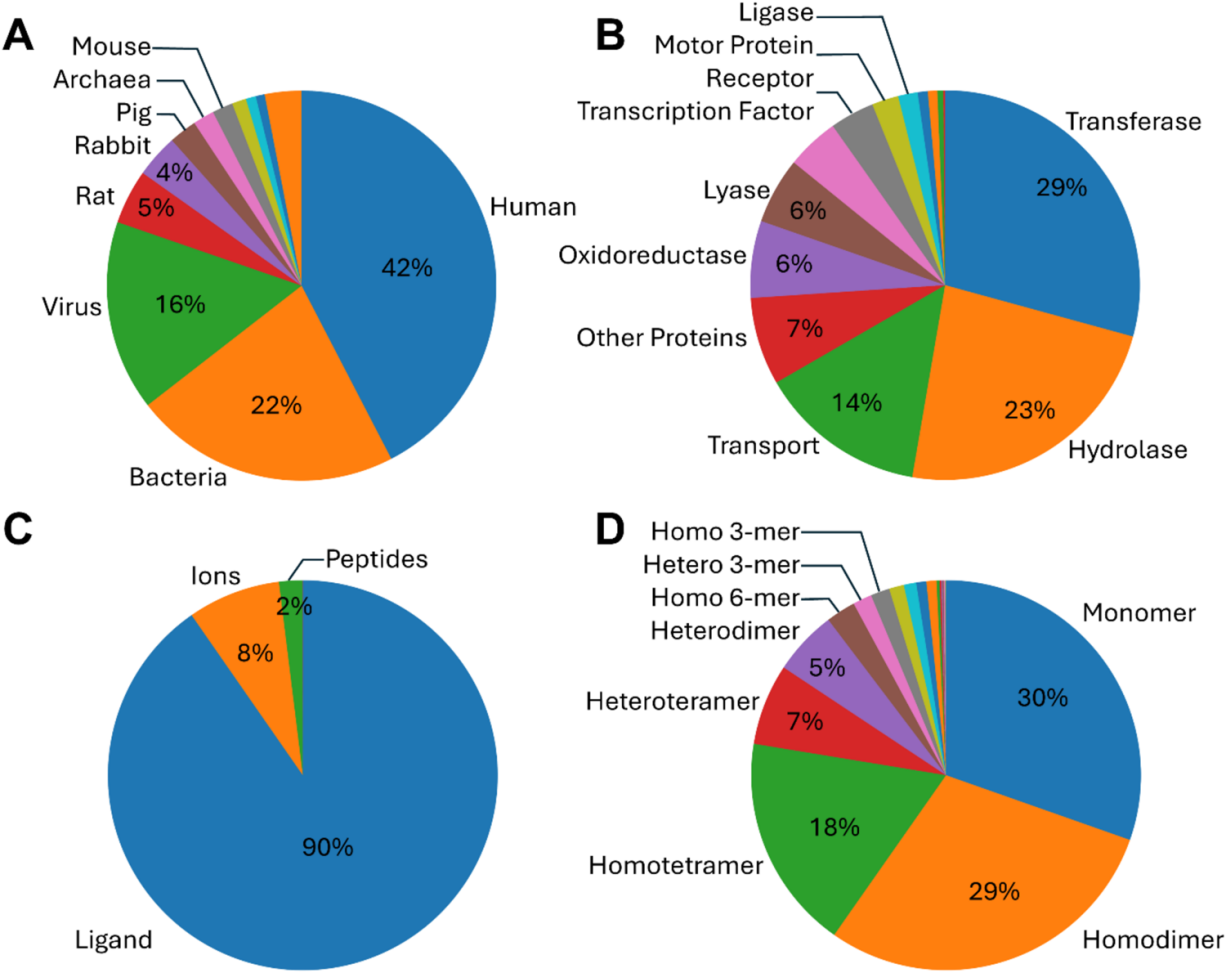
Types of protein in AlloBench according to (A) source organism, (B) function, (C) type of allosteric modulator, and (D) oligomeric state. The complete data is provided in Tables S4–S7.

AlloBench is a data set creation pipeline for allosteric proteins with known allosteric and active site residues in proteins. The proteins in the data set are primarily from humans, bacteria, and viruses, and they perform various functions (Figure 4A,B). It uses the ASD as a starting point and adds the active site residues from UniProt and M-CSA. It further enriches the data by fetching and incorporating the protein name, review status, UniRef clusters IDs, and sequence from the UniProt, and oligomeric state, experimental method, structure resolution, and the mapping from the chains to UniProt IDs from the PDB. Finally, AlloBench outputs a CSV file with all this information, most notably the list of allosteric and active site residues. It also downloads the PDB structures, ensures the annotation of the allosteric and active site residues agrees with the residue numbering in the PDB file, and fills in the missing residues.

The AlloBench creates a data set of 2141 allosteric sites from 2034 PDB structures containing 418 unique UniProt chains. While almost all proteins have only one allosteric modulator, 61 structures with the same PDB ID bind to 2 different allosteric modulators. 90% of the allosteric sites bind to small molecule ligands, 8% bind to ions, and only 2% have peptides as allosteric modulators (Figure 4C). 83% of the data set is comprised of homomeric proteins, and 17% are heteromeric (Figure 4D).

### Relative Performance of Allosteric Site Prediction Tools

**Figure 5:**
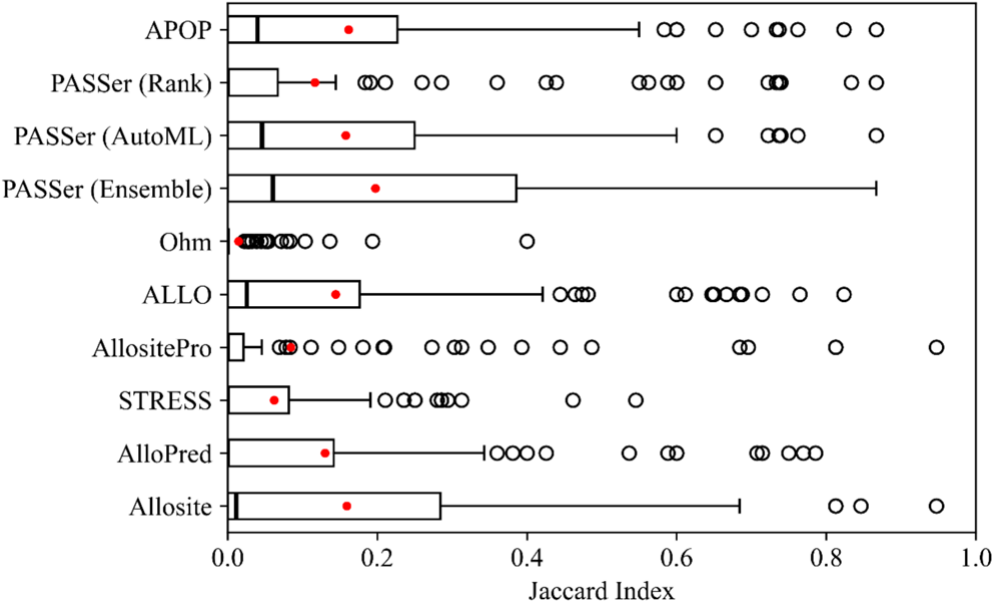
Jaccard index of each program/web server’s topmost predicted allosteric site; the higher values are better. The medians and means are shown in black bars and red dots, respectively. Ohm does not rank its predicted allosteric sites, so only the first prediction is considered here.

The distributions of JI for the topmost predicted sites from each program are depicted in Figure 5, and the histograms of JI for all predicted sites from each program are provided in Supporting Information Figure S2. The distributions for monomeric proteins (with multimers excluded) are presented in Figure S3. Surprisingly, we found that most predictions from the programs had no overlap with the known allosteric site, as evidenced by the high frequency of JI = 0 in each subplot of Supporting Information Figure S2. The trend holds true for the distribution of JI of the topmost prediction from each program, as the median is close to 0 in all cases in Figure 5. It also shows that PASSer (Ensemble) performs the best with a median JI of 0.060 (and a mean of 0.197), followed by PASSer (AutoML), APOP, ALLO, and Allosite with a median of 0.046, 0.040, 0.025, and 0.011, respectively. For highly skewed distributions like the ones in Figures 5 and S2, the median is a better measure of central tendency than the mean, as outliers do not influence it to the same extent as the mean. However, the remaining programs, AlloPred, PASSer (Rank), AllositePro, STRESS, and Ohm, all have a median of 0 and thus can only be ordered by their mean Jaccard indices of 0.130, 0.117, 0.084, 0.062, and 0.014, respectively.

AllositePro could not find the allosteric site for 35 proteins (see Supporting Information Table S3), and a JI of 0 was assigned to these cases, explaining its poor performance. Allosite could not find the allosteric site for 7 proteins, but that did not adversely affect its performance to the same extent. The poor performance of STRESS may be attributed to its predictions being limited to 10 residues per site.^23^ Ohm is the only method completely free of pocket detection. If we consider the allosteric site prediction with the maximum JI from Ohm as its topmost prediction instead of the first prediction for each protein, the median becomes 0.129 and 0.164 for the mean. This indicates that Ohm can find a hotspot close to the allosteric site among its numerous hotspots. However, the lack of ranking the hotspots diminishes its utility, as the user does not know the best site a priori. The JI of the topmost predicted sites from each tool for the 100 proteins in the test set is listed in Supporting Information Table S3, which shows that there are very few proteins for which all tools performed equally well.

### Accuracy of Allosteric Site Prediction Tools

**Figure 6:**
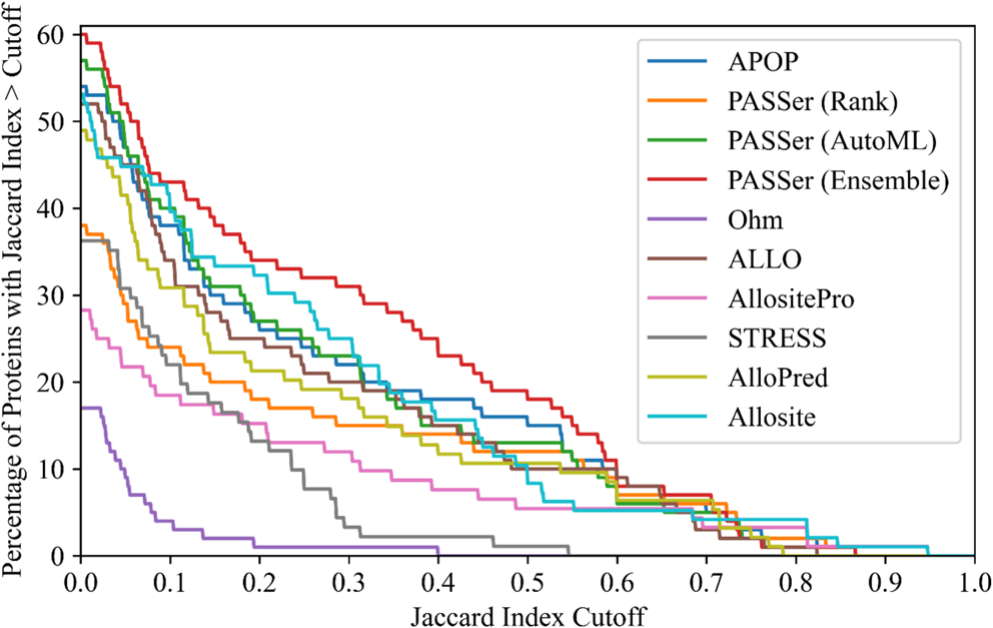
Percentage of proteins with a Jaccard index (JI) above the cutoff for the topmost allosteric site prediction from each program for the 100 proteins in the test set. Ohm does not rank its predicted allosteric sites, so the first prediction is considered here. The JI cutoff is given on the *x*-axis. The detailed values are provided in Supporting Information Table S8.

To estimate the accuracy of these programs, we calculated the percentage of proteins in their top predictions with JI larger than a varying threshold (Figure 6). Surprisingly, none of these programs could achieve an accuracy of more than 60%, even with a very low JI cutoff of approximately zero. Suppose we consider correct predictions to be those with a JI > 0.5. The top three programs are PASSer (Ensemble), APOP, and PASSer (AutoML), with an accuracy of 18, 15, and 13%, respectively. Note that choosing a different threshold affects the accuracy estimates quantitatively and, thus, the order of the programs in terms of accuracy. Still, PASSer (Ensemble) has the highest accuracy for almost all the cutoff values. The accuracy estimates for the programs at cutoffs of 0, 0.1, 0.2, 0.3, 0.4, and 0.5 are tabulated in Supporting Information Table S8. It is noteworthy that although the performance of the tools appears poor when calibrated using the known allosteric sites, the tools might be detecting novel allosteric sites yet to be annotated, and the allosteric sites obtained from ASD and the active sites obtained from UniProt and M-CSA may have incomplete or incorrect residue annotation.

**Figure 7:**
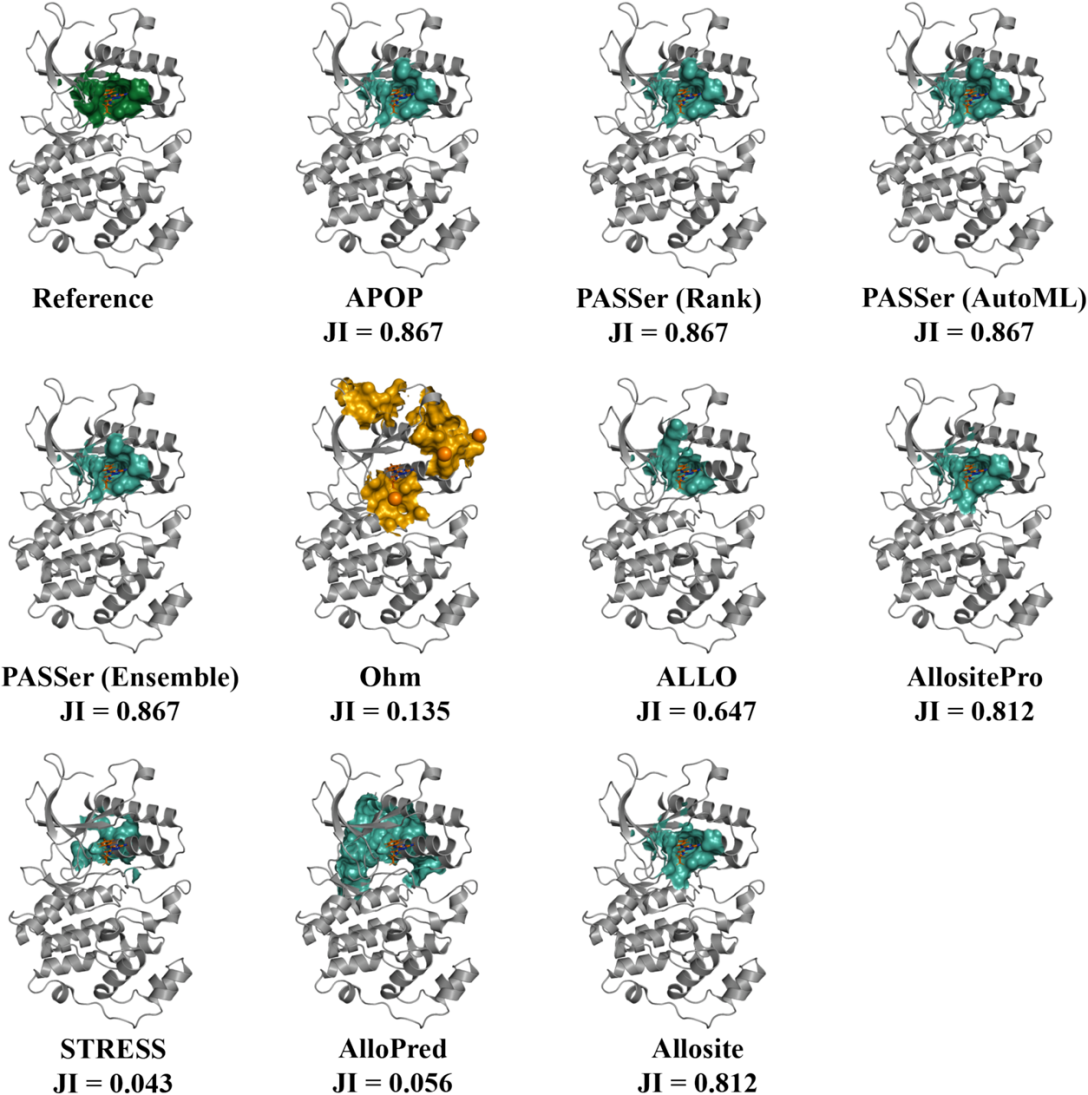
Top-ranked predicted allosteric sites in Human Mitogen-activated protein kinase 7 (PDB ID: 4ZSG) from each program and web server. The Jaccard index (JI) of each prediction is given below the respective structure. In the reference structure, the known allosteric site is colored dark green. The predicted allosteric sites are colored green. The location of all Ohm-predicted hotspots is shown with orange spheres, and the surface residues within 8 Å are shown in orange. As Ohm does not rank its hotspots, the JI of the best prediction is provided. The allosteric modulator is shown in the licorice model.

To better understand the accuracy of various tools, the top-ranked prediction from each for Human Mitogen-activated protein kinase 7 is visualized in Figure 7. These predictions matched the known allosteric site well, except for AlloPred and STRESS. The predicted allosteric site from APOP and the three models of PASSer are identical, likewise for the predictions from Allosite and AllositePro. Identical residues are invariably returned from these programs as allosteric sites because most use Fpocket to identify the protein surface pocket. The slight difference between the allosteric sites from APOP/PASSer and those from Allosite is ascribed to different Fpocket input parameters, versions, or both. Additional examples are demonstrated in Supporting Information Figures S4 and S5.

### Speed of the Allosteric Prediction Tools

In addition to the accuracy, we also tried to calibrate the speed of these tools. Most allosteric site prediction tools are available exclusively as web servers, and the source code or offline programs are unavailable. Therefore, it was impossible to quantitatively benchmark the speed of each tool on the same computer hardware. Qualitatively, we found most tools to return their predictions within seconds to minutes, except for AlloPred and STRESS, which ran for hours on some larger proteins as they performed multiple NMA calculations.

## Discussion

The information on allosteric sites in proteins is scarce, and data sets curating allosteric proteins are invaluable. There are over 250 million sequences of known protein chains in the UniProt, and only 65,855 sequences have annotations for allosteric sites. Of these 65,855 sequences, only 676 have structures available in the PDB. The lack of structures solved with bound substrates and allosteric modulators adds to the challenge of accurately identifying the allosteric and active sites. ASD complements this with almost 3000 structures bound to allosteric modulators in a rich interactive web database. The data sets, such as ASD, ASBench, and CASBench, were developed from the perspective of curating individual allosteric sites and maximizing the diversity of the data set by providing a representative structure for each protein or allosteric site. However, data about the same allosteric site in different proteins is still valuable, especially for data-hungry applications such as AI. The data processing requirement might be case dependent. For example, allosteric site prediction tools accept protein structures (and the active site residues in some cases) as input, while the output is the list of allosteric site residues. Hence, we designed AlloBench with a structure-first approach focusing on easy accessibility to the list of active and allosteric site residues to enable the testing and benchmarking of such tools.

Interestingly, finding the number of proteins in the data sets proved to be a nontrivial task, as the biological assembly of proteins does not have unique identifiers. UniProt IDs correspond to protein chains, whereas PDB IDs are used for biomolecular structures. The same protein in different states or bound to different ligands has different PDB IDs. For homomeric proteins, the UniProt ID is a unique identifier of biologically active proteins, but for heteromeric proteins, these do not uniquely correspond to proteins in vivo. For example, human hemoglobin is heterotetrameric, comprised of two α-chains (UniProt ID P69905) and two β-chains (UniProt ID: P68871). Therefore, the same protein PDB ID contributes two UniProt IDs. Hence, we report the number of rows, UniProt IDs, and PDB IDs to better estimate the number of biologically active proteins.

Often, multimeric proteins have the same allosteric and active sites on all identical chains. In addition, the allosteric or active sites at the interface of multiple chains may manifest only in biological assembly structures. It is important to provide the biological assembly structure of multimeric proteins to allosteric site prediction tools as potential allosteric sites on the surface of a single chain may be buried at the interfaces of the chains, making the predicted allosteric site inaccessible to the allosteric modulator. Similarly, the missing residues in PDB structures may also contribute to allosteric sites. The same protein binding to multiple substrates or modulated by multiple allosteric modulators is often overlooked. Such cases add to the complexity of studying allostery in proteins, and such information is often lacking in allosteric databases. The association between the substrate, active site, the allosteric modulators, and the allosteric site must be captured in these proteins.

Two aspects must be considered to predict allosteric sites: (i) the site must enable the binding of the allosteric modulators, and (ii) the binding of the modulator at the site must perturb a distant active site. Most programs focused on the first aspect, and only a few considered the second one. All programs except Ohm first use a pocket detection tool to identify candidate pockets on the protein surface. Then, AI models trained on the pocket features or perturbation-based scoring functions (Figure 2) rank or classify the sites as allosteric. All pocket-based allosteric site prediction tools, except for ALLO and STRESS, used Fpocket as it is fast, easy to use, integrates easily with other software, and is actively maintained. Hence, these tools share the same limitation of the accuracy of Fpocket. Many other pocket-detection tools are available, like SAPocket,^33^ ScanNet,^41^ PUResNet,^42^ DeepBindPoc,^43^ P2Rank,^44^ and DeepSite.^31^ These may be combined with existing or novel algorithms classifying or ranking pockets into allosteric sites to improve performance. Pocket detection tools are primarily designed to predict the binding sites of small drug-like molecules. So, allosteric site prediction programs relying on these tools will likely underperform when detecting sites for other kinds of allosteric modulators, such as gases, ions, peptides, proteins, and nucleic acids. Thus, more pocket-free methods like Ohm are desired.

The perturbation-based methods incorporate dynamic information, generally performing NMA of bound and unbound states of the protein and quantifying the global change from the binding of the allosteric modulator. AlloPred and Ohm are the only programs that explicitly check whether the binding of the allosteric modulator at the predicted site explicitly perturbs the active site. This requires the input of the active sites, which is known for only 18% of the proteins in UniProt. Solely relying on the fluctuation propagation like Ohm also results in poor performance. Therefore, a combination of surface features and perturbation propagation is required, as used by APOP.

Surprisingly, PASSer (Ensemble) surpassed the sophisticated models provided by PASSer (AutoML and Rank). This is likely because it combines two models, one learning the structural properties and the other learning pocket features, as opposed to the models trained solely on pocket features. An ensemble of models trained on pocket features, structural information, and perturbation propagation is warranted. The test set employed here was created such that proteins in or related to those in the training sets of allosteric site prediction tools were excluded. Thus, any mismatch between AlloBench and PASSerRank/AutoML training sets is included in the test set. AlloBench is a significantly larger and updated data set than the training set employed by PASSer Rank/AutoML and other tools. Retraining the allosteric site prediction tools using training, validation, and test splits created from AlloBench would substantially improve their performance.

Existing allosteric site prediction programs use ML algorithms like support vector machine, random forest, and XGBoost, whereas the application of deep learning is mainly unexplored. A recent deep-learning study on the structural changes of proteins for the prediction of the orthosteric and allosteric sites demonstrates the latter to be more challenging.^45^ However, advances are underway to develop computational protocols to predict the binding structure of protein multimers and those that detect cryptic pockets in proteins.^46,47^

In addition to the programs/web servers explored in Table 1, many other tools exist that could not be included in this study. For instance, the PARS web server^48^ encounters software errors after job submission, and no active link or source code is available for MCPath^49^ and SPACER.^50^ Programs that do not provide any web server or software implementation were also excluded, such as TopoAlloSite,^51^ Essential Site Scanning Analysis (ESSA),^52^ and those using bond-to-bond propensities^53^ or a random forest model.^54^ Many such tools are now defunct since they were only available as web servers, and no binaries or source code were provided. The availability of source code would also foster transparency and promote future development without having to rewrite many algorithms and tools. If sharing the source code is not possible, at least archiving the binaries would prevent the tools from becoming obsolete. API to web servers and downloadable binaries or source code also facilitates the local execution for large-scale prediction tasks. We took the additional effort of installing most of the tools locally and running the predictions in bulk for completeness. Investing time and effort in installing and running these programs locally is inadvisible for individual predictions as they do not offer a significant advantage over user-friendly web servers like APOP, Allosite, and PASSer.

## Conclusions

The AlloBench is a high-quality data set of allosteric proteins with allosteric and active site information enriched by additional data from the UniProt and PDB databases that is suitable for computational studies. The associated pipeline can update the data set with the latest database information. We also compared the performance of the allosteric site prediction tools. We show that the Jaccard index can be used to quantitatively evaluate the performance of all allosteric site prediction tools examined here. The overall performance of most programs was found to be well below 60%, with PASSer (Ensemble) outperforming the rest. In addition to novel approaches to allosteric site prediction, more reliable data on allosteric sites of proteins is needed. As allosteric and active site information continues to grow, the pipeline for data filtering and refinement presented here will not only enable the development of improved allosteric site prediction tools but also the study of allosteric proteins using traditional and AI approaches alike.

## Data Availability

The AlloBench data set pipeline is available at https://github.com/djmaity/allobench. It is open-source and written in Python. Detailed installation and run instructions are provided in the repository. Additional analysis scripts and Jupyter notebooks in Python are available at https://github.com/djmaity/allostery.

## Supporting Information Available

Summary of allosteric site prediction tools, and details of how to run these tools; identifying proteins in the test set of allosteric prediction tools using AI; supporting figures on the relation between the JI and the distance between the predicted and experimental allosteric sites, distribution of the JI, and structure of top-ranked predictions; supporting tables of features of AlloBench and other packages, JI and distributions of the 100 proteins in AlloBench (PDF)

## Abbreviations

ASD: AlloSteric database
API: application programming interface
AutoML: Automated machine learning
CASBench: catalytic and allosteric sites benchmark set
M-CSA: mechanism and catalytic site atlas
PDB: protein data bank
JI: Jaccard Index
NMA: normal-mode analysis
