# Supplementary material for: AlloBench: A Data Set Pipeline for the Development and Benchmarking of Allosteric Site Prediction Tools
Source: ACS Omega. 2025 Apr 23;10(17):17973–82. doi: 10.1021/acsomega.5c01263 (PMC12059942; doi:10.1021/acsomega.5c01263)
Supplement: Supplementary file 1 — ao5c01263_si_001.pdf [file ao5c01263_si_001.pdf]

Supporting Information

AlloBench: A dataset pipeline for the development and benchmarking of allosteric site prediction tools

Dibyajyoti Maity and Baofu Qiao*

Department of Natural Sciences, Baruch College, City University of New York,

New York, New York 10010, United States

Email: baofu.qiao@baruch.cuny.edu

S1. Summary of Each Allosteric Site Prediction Tool

Allosteric Pocket Prediction (APOP).

APOP is AI-free and the only program studied here that combines pocket detection using Fpocket with NMA. First, the pockets in the protein are identified using Fpocket. A Gaussian network model of the protein is constructed, and the normal modes are evaluated. Next, for each predicted pocket, the NMA of the protein is repeated using a higher spring constant connecting residues of the pocket to model the perturbation from modulator binding at the candidate pocket. This process is similar to AlloPred till this step, except that a Gaussian network model is used in APOP in contrast to an elastic network model in AlloPred. The 5 out of the 15 slowest normal modes of the perturbed system are matched to the 5 slowest normal modes of the unperturbed system. After matching the normal modes between the two systems, the average percentage change in the corresponding eigenvalues is calculated. Finally, the pockets are ranked using a score that is the average of the z-score of this eigenvalue change and the z-score of local hydrophobic densities obtained from Fpocket.

PASSer (Protein Allosteric Sites Server).

The PASSer web server provides three ML models: Ensemble, AutoML, and Learning-to-rank (Rank). These models were trained on 19 pocket features from the Fpocket program (Supporting Information Table S2). The Ensemble learning and AutoML models report predicted probabilities of the top 3 pockets, while the Rank model reports rank scores. In addition to the interactive web server, PASSer provides a convenient Application Programming Interface for running multiple jobs.

a) Ensemble: XGBoost + Graph Convolutional Network. The ensemble model combines an XGBoost (eXtreme Gradient Boosting) model trained on Fpocket features and a Graph Convolutional Network trained on the atomic graph for each pocket to learn the local connectivity. The average probabilities from the two models indicate the likelihood of the pocket being an allosteric site.

b) Automated machine learning (AutoML). AutoML automates ML pipelines, which include tasks such as data preparation and preprocessing, feature engineering, model selection, and hyperparameter tuning. The PASSer's AutoML model was developed using the AutoGluon framework (v0.2.0). This framework creates a multilayer stack ensemble of 13 models, including k-nearest neighbors, gradient-boosted decision trees, random forests, and neural networks. Hence, it is computationally slower than the Ensemble and Rank models.

c) Rank (Learning-to-rank). Rank makes "relative" predictions by ranking the pockets detected by Fpocket according to the possibility of being an allosteric site in contrast to the other models that perform an independent "absolute" evaluation of the probability of each pocket being an allosteric site. The LightGBM framework was used to train a LambdaMART model consisting of gradient-boosted decision trees with the LambdaRank objective function.

Ohm

The Ohm web server is primarily designed to analyze allosteric communication networks and pathways solely based on the protein structure using a stochastic perturbation propagation algorithm. It also claims to predict allosteric sites based on the active site, but the web server only returns the allosteric coupling intensity of the residues in the protein. Allosteric coupling intensity is the normalized value of the number of times a residue propagates a perturbation from the active site. The allosteric hotspots can be identified by traversing the connectivity network of the protein

constructed based on a distance cutoff and searching for local maxima of allosteric coupling intensity among the nodes. The hotspot detection algorithm is only available through the source code but is not implemented on the web server. The allosteric hotspots are points in 3D space with respect to the input structure and do not explicitly list the residues in the predicted site. Unfortunately, the hotspots are not ranked as opposed to the outputs from other allosteric site prediction tools.

ALLO

ALLO provides two ML models implemented in Python trained on the descriptors from DoGSiteScorer, a pocket detection program. The first is a Naïve Bayes classifier that classifies the pockets as an allosteric or orthosteric site. The second is an artificial neural network that returns the probability of the pocket being an allosteric site. The allosteric sites identified by the Naïve Bayes classifier can be ranked using the probability from the artificial neural network to find the top-ranked allosteric site predictions.

Allosite and AllositePro

Allosite was the first user-friendly allosteric site prediction web server created by the same development team of ASD. It provides two independent methods, Allosite and AllositePro. Allosite, released in 2013, uses a support vector machine classifier trained on the 21 features (Supporting Information Table S2) of pockets identified by Fpocket in 90 non-redundant allosteric proteins from ASD. Later, AllositePro was introduced, which uses a combination of logistic regression and perturbation analysis using NMA. The logistic regression model was trained on three features from the Fpocket program: normalized maximum distance between two α-spheres, normalized hydrophobicity density, and flexibility score. Meanwhile, the NMA was done for the protein with and without a dummy ligand bound to the site predicted by Fpocket. The dummy ligand consists of 7 points obtained by first generating the α-spheres using Fpocket, clustering the α-spheres into 7 clusters by distance, and calculating the center of each cluster. The B-factors of the atoms were calculated from the NMAs, and the p-value of the Mann–Whitney U-test for the two B-factor distributions was then obtained. The score from the perturbation method (PNMA) is PNMA = 0.05/(0.05 + p-value). The probability of a pocket being an allosteric site is consequently characterized using 0.8 × Pfeature + 0.2 × PNMA, where Pfeature is the probability from the logistic regression model.

STRESS

STRESS can identify surface critical residues (potential allosteric sites) and interior critical residues (responsible for transmitting allosteric perturbation through protein structure). To identify potential allosteric sites, it probes the protein surface for pockets where a pseudo ligand comprised of four atoms can sit using Monte Carlo simulations. The list of residues in the site is ordered by local closeness, a measure based on the degree of nodes in the residue-residue contact network constructed from the protein. The site with the highest local closeness is selected after each Monte Carlo simulation. The sites from multiple Monte Carlo simulations with considerable overlap are merged, resulting in a list of candidate sites on the protein surface. Next, the binding leverage score is calculated by the sum of the squared displacement between pairs of residues in each of the 10 slowest normal modes from NMA of the protein structure. The candidate site can be ranked using the binding leverage score to obtain the top predicted allosteric sites. Note that the STRESS output is limited to 10 residues per allosteric site.

AlloPred. AlloPred uses Fpocket to predict pockets on the protein surface. Then, the unperturbed and perturbed NMA is done for the protein using an elastic network model. The perturbation is simulated using a higher spring constant for connections involving the pocket residues. Next, the influence on the active site residues is quantified by averaging the absolute difference of displacements from equilibrium in the perturbed versus the unperturbed NMA and scaling the average with the normal mode frequency. Thus, AlloPred requires the active site residues as input. In addition, the perturbation effect per residue was also calculated. These and the Fpocket features listed in Supporting Information Table S2 were used to train a support vector machine model.

S2. Identifying Proteins in the Test Set of Allosteric Prediction Tools using AI.

The PDB IDs for the 90 proteins from ASD used to train Allosite and PASSer Ensemble were obtained from the supporting information of the respective publications, but the corresponding UniProt IDs were absent. The obsolete PDB IDs were identified and replaced with the superseded PDB IDs. For each PDB ID, the UniProt IDs of the chains in the structure were obtained using PDB's GraphQL API. A PDB structure may contain multiple chains, each with its UniProt IDs; hence, finding the correct UniProt ID is not straightforward. For each PDB ID, if the corresponding UniProt ID in the ASD was found in the list of UniProt IDs from PDB, then that UniProt ID was assigned to the PDB ID. For 3 PDB IDs (3PJG, 3BEO, 3HO8), the UniProt ID from ASD did not

match the only UniProt ID fetched from the PDB. The UniProt ID from PDB was considered the correct and updated one in these cases. 16 PDB IDs (1ESM, 1KP8, 2X1L, 2OI2, 1QTI, 3GCP, 3OS8, 2QF7, 1COZ, 3FIG, 2GS7, 1DB1, 1RX2, 2BND, 1XLS, 3IDB) were not found in ASD. Among them, the first 14 had only one UniProt ID fetched from the PDB for each structure, which was assigned to the PDB ID as the correct UniProt ID. Identifying the appropriate UniProt ID for the PDB IDs 1XLS and 3IDB was not possible, so all identified UniProt IDs were assigned to these two PDB IDs.

Similarly, the training data for ALLO is available in its GitHub repository, and the PDB ID of the proteins was extracted from the 'name' column. The PDB IDs of the 207 proteins used to train the PASSer's AutoML and Rank models are unavailable. The data processing pipeline is available at https://github.com/smu-tao-group/PASSerRank, and the processed dataset is available at https://passer.smu.edu/static/allorank.zip. We modified the data processing pipeline to output the PDB IDs of the processed structures in addition to the pocket features. We matched them to the features in the downloaded dataset in the allorank.zip file. We could identify the PDB IDs for 206 of 207 proteins in the dataset. We followed the process described above to replace obsolete PDB IDs and obtain the UniProt IDs corresponding to the PDB IDs. The UniProt ID L8DYY9 for the PDB ID 4QSK is no longer in the UniProt and was dropped from the dataset.

AllositePro was trained using the ASBench Core-Diversity set, and the AlloPred training set is available from AlloPred's GitHub repository. Both have the appropriate UniProt IDs assigned to the proteins' PDB IDs. The sets were also subjected to removing obsolete PDB IDs and updating the UniProt ID as described above.

S3. Running Allosteric Site Prediction Tools

APOP

Running APOP smoothly on Linux and only requires the dependencies numpy, fpocket, and py-packman to be installed, which is easily accomplished in a Python conda environment with:

```
conda install -c conda-forge numpy fpocket
pip install py-packman
```

APOP prediction can be run with the following:

```
python apop.py input.pdb
```

This creates an archive with the structure of the protein in a PDB file, the atoms of each pocket in separate PDB files, and an output text file with the rank, name of the corresponding PDB file with the atoms of the pocket, the APOP score and the residues of each pocket.

PASSer (Ensemble, AutoML, and Rank)

The PASSer web server provides an API where allosteric sites can be obtained by uploading the PDB file with the following command:

```
curl -X POST -F pdbFile=@filename.pdb -F model=ensemble https://passer.smu.edu/api
```

Here, the AutoML and Rank models can be specified with the `model=automl` and `model=rank`, respectively. The residues of the top 3 predicted allosteric sites are returned in JSON format, along with their probabilities for ensemble and autoML models. The rank scores are provided for the rank model. The allosteric site residues were reported using VMD (Visual Molecular Dynamics) selection language, which was converted to the form of chain-3_letter_amino_acid_code-residue_number. Occasionally, PASSer returned unusual chain identifiers and residues for some cases. For example, The output of PDB ID 5OS2 had entries in the form "(chain 1 and resid -18.156 -18.4 74)". Such residues were excluded from the predicted allosteric sites.

Allosite and AllositePro

The Allosite and AllositePro web server hosted on the same webpage provides no offline tool or source code to run the predictions locally. Hence, the structure files from the test set were manually submitted, and all the chains were selected for consideration. The prediction reports were downloaded as archives containing three files: one .pdb file from the Fpocket run containing the protein structure and pseudo atoms representing the pockets, one PyMOL .pml file for visualizing the pocket residues, and one text file with the values of the various features and scores. The residues of the predicted allosteric pocket were parsed from the `.pml` files.

Ohm

Ohm was run locally as the hotspot identification script is exclusively available in the source code. To install Ohm use the following installation instructions that have been tested on the Linux operating system Ubuntu 22.04 and 23.04.

1. Clone the Ohm repository

```
git clone https://bitbucket.org/dokhlab/ohm.git
```

2. Install the dependencies

```
sudo apt install zlib1g-dev
```

3. Go to the cloned directory, compline and install Ohm

```
cd ohm
echo '/path/to/install/location'>.prefix
make -j16
make install
```

4. Add Ohm to `${PATH}`. These commands may be used each time before running Ohm or added to the `${HOME}/.bashrc` file for permanent access to Ohm commands

```
export PATH="/path/to/ohm/bin:/path/to/ohm/scripts:$PATH"
export OHM_HOME="/path/to/ohm/"
```

5. Fix the Ohm script to run with Python 3.

Suppress the module load commands in the shell scripts with

```
sed -i 's/^module load/# module load/g' *.sh
```

Change the strings in regular expression calls to literal strings in Python scripts

```
sed -i 's/re.split(/re.split(r/g' *.py
```

Change line 59 in plot-nodes.py from plt.colorbar(plot) to plt.colorbar(plot, ax=plt.gca())

```
sed -i 's/^plt.colorbar(plot)/plt.colorbar(plot, ax=plt.gca())/g' plot-nodes.py
```

Change bfactor1 to bfactor in hotspots.sh

```
sed -i 's/bfactor1/bfactor/g' hotspots.sh
```

6. Ohm requires the following Python packages to run its scripts: *cmocean*, *colour*, *matplotlib*, *numpy*, and *scipy*. We highly recommend installing these in a virtual environment, preferably using the Anaconda (https://www.anaconda.com) or Miniconda (https://docs.anaconda.com/miniconda/) Python distribution, as shown below.

```
conda create -n ohm
conda activate ohm
conda install -c conda-forge cmocean colour scipy matplotlib numpy
```

The input parameters to Ohm were specified using a bash script, as shown below

```
name="1A3W"
active="A/49,A/51,A/52,A/53,A/54,A/84,A/85,A/91,A/177,A/240,A/242,A/265,A/266,A/298,A
/402,A/403,A/404,A/405,A/406,A/407,A/452,A/459,A/484,B/49,B/51,B/52,B/53,B/54,B/84,B/
85,B/91,B/177,B/240,B/242,B/265,B/266,B/298,B/402,B/403,B/404,B/405,B/406,B/407,B/452
,B/459,B/484"
alpha="4.5"
distance_cutoff="3.4"
probability_cutoff="0.05"
rounds="10000"
pathways="100"
cell_size="1"
volume_radius="5"
volume_ratio="0.3"
pockets=50
vdw="3.3"
shell="0.1"
```

Only the name and active site residues were changed for each case, and the rest of the parameters retained the default values. First, the ACI calculation script (aci.sh) was run to obtain a PDB file with the ACI values in the B-factor column. Next, the PDB file was passed to the hotspots command that outputs another PDB file with pseudo atoms at the 3D coordinates of each hotspot. The residues within 8 Å of each hotspot were identified using PyMOL and considered the allosteric sites predicted by Ohm. Various cutoff distances were tried and visually inspected to select 8 Å as the suitable cutoff distance.

ALLO

ALLO requires the output from DoGSiteScorer. But the output from the latest version of the DoGSiteScorer on the ProteinsPlus web server (https://proteins.plus/help/dogsite) is incompatible with ALLO. DoGSiteScorer 2.0 from https://www.biosolveit.de/academic-drug-discovery/#DoGSiteScorer had thus to be used. It was run with the following command:

```
dogsite -p input.pdb -o output_name -d -w 3 -i
```

'-d' specifies to write the pocket descriptors to an output file, which is the input for ALLO, '-w 3' instructs the program to write the pocket amino acids, and '-i' specifies to write the druggability scores into the descriptors file. The Naïve Bayes classifier and the Artificial Neural Network (ANN) were run on the DoGSiteScorer descriptors files with the following commands.

```
python predict_nb.py output_name_desc.txt
python rank_nn.py output_name_desc.txt
```

The output from the Naïve Bayes classifier is one text file with three columns: the prediction label 'O' for orthosteric and 'A' for allosteric, the pocket name, and a score > 1 for orthosteric pockets and < 1 for allosteric pockets. The output from the ANN is again a text file with the name of each pocket and the probability of the pocket being an allosteric site. The pockets predicted as allosteric by the Naïve Bayes classifier were ranked using the probability from the ANN. The residues of the predicted site were obtained from the corresponding PDB files containing the pocket atoms.

STRESS

STRESS depends on the Molecular Modelling Toolkit (https://github.com/khinsen/MMTK), which hasn't been updated in years and requires Python 2. The installation of MMTK was challenging on current Linux distributions. As a workaround, MMTK and STRESS were installed on an Ubuntu 14.04 virtual machine, which was easily accomplished with `'sudo apt install python-mmtk'` and following the installation instructions in the source code for STRESS. The surface-critical residues were evaluated with STRESS using the following command:

```
./stress.sh -I input.pdb -O output_directory -L logfile.txt -s
```

AlloPred

The AlloPred web server is no longer operational, so the source code was downloaded and run locally. The requirements for both Fpocket and AlloPred could not be satisfied simultaneously and were installed in separate virtual environments. First, the Fpocket was run to detect the pockets. AlloPred was run from a directory containing the protein structure (input.pdb), the output directory from Fpocket, and the active site residues specified in a text file (act_res.txt).

```
python path/to/run_allopred.py input.pdb_directory act_res.txt
```

The predicted allosteric site residues are in the output text file.

S4. Supporting Figures

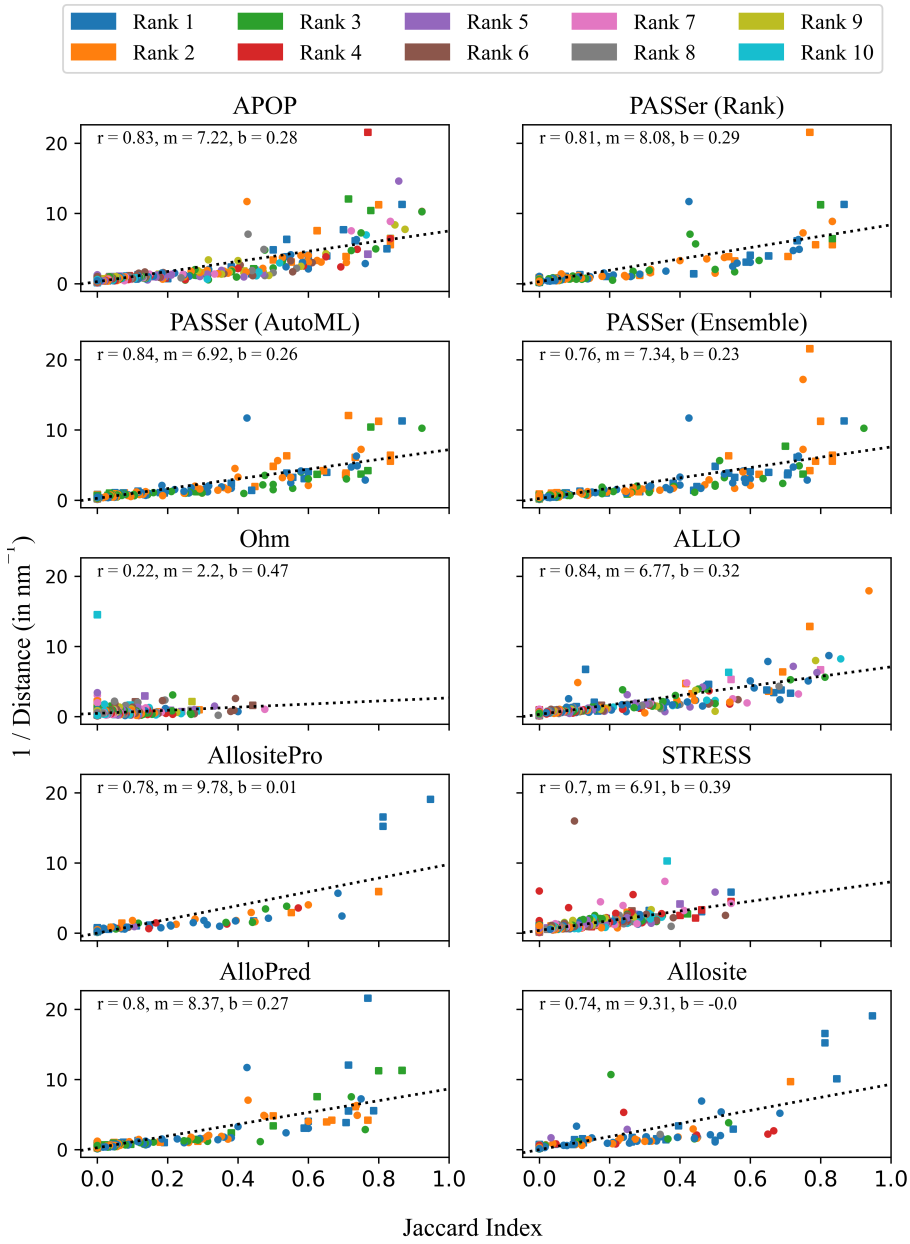

Figure S1: The Jaccard Index (JI) *vs.* inverse of the distance between the known allosteric site and the predicted site from each program or web server. Up to the top 10 predictions for each protein are depicted. The results for monomeric and multimeric proteins are represented by square and circular markers, respectively. The r in each subplot is the Pearson correlation coefficient, while m and b are the slope and intercept of the regression line. The strong correlation between JI and the inverse of the distance indicates that JI can estimate the proximity between the known and predicted sites. However, the variability at high JI and small distances suggests that the mere proximity of the allosteric sites is insufficient to ensure proper overlap, as the centroid of the sites may be close together, but the orientation of the pocket (residues of the sites) may differ.

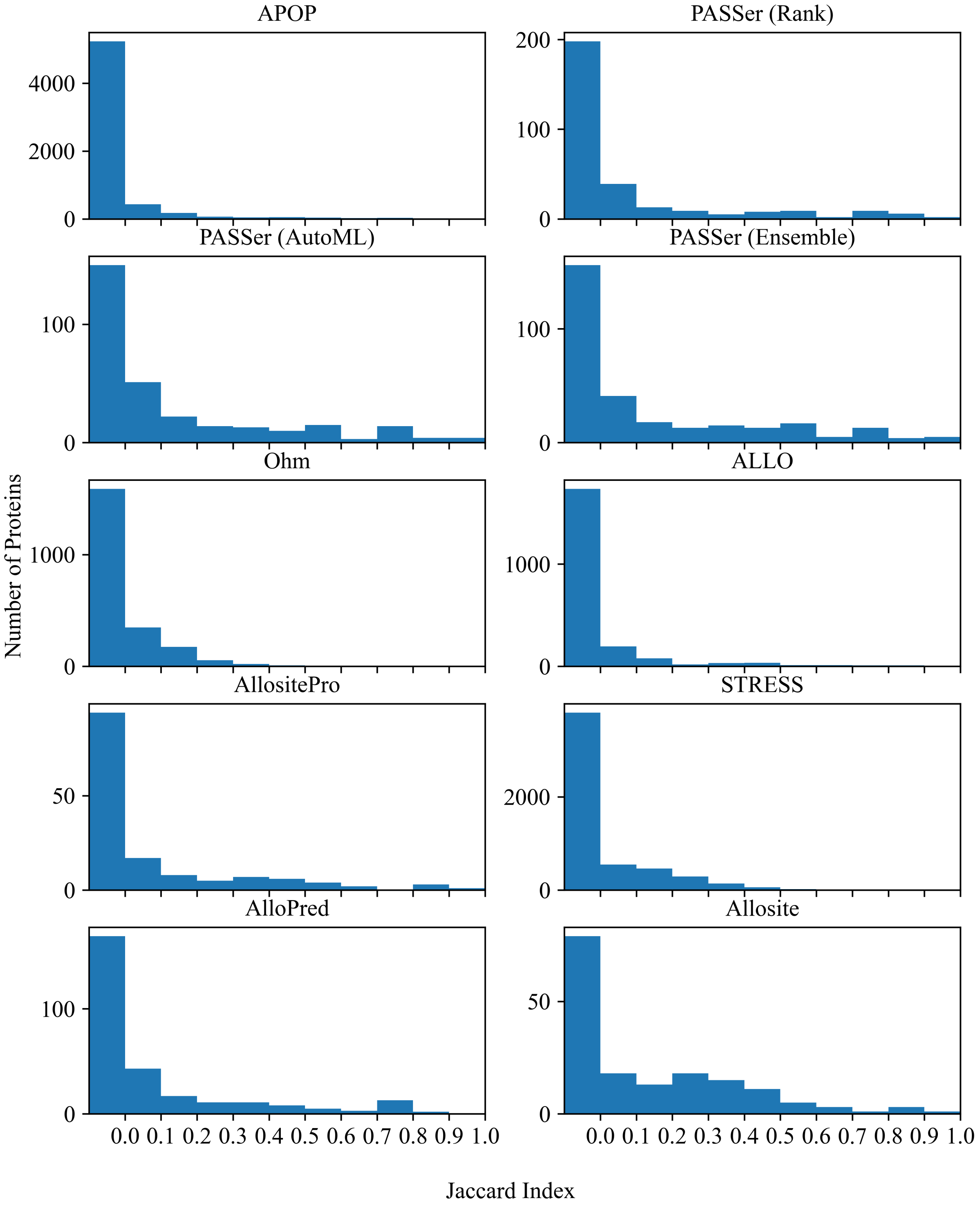

Figure S2: Histogram distribution of Jaccard Index of all predicted allosteric sites from each allosteric site prediction tool. The first bar is for the Jaccard Index = 0.

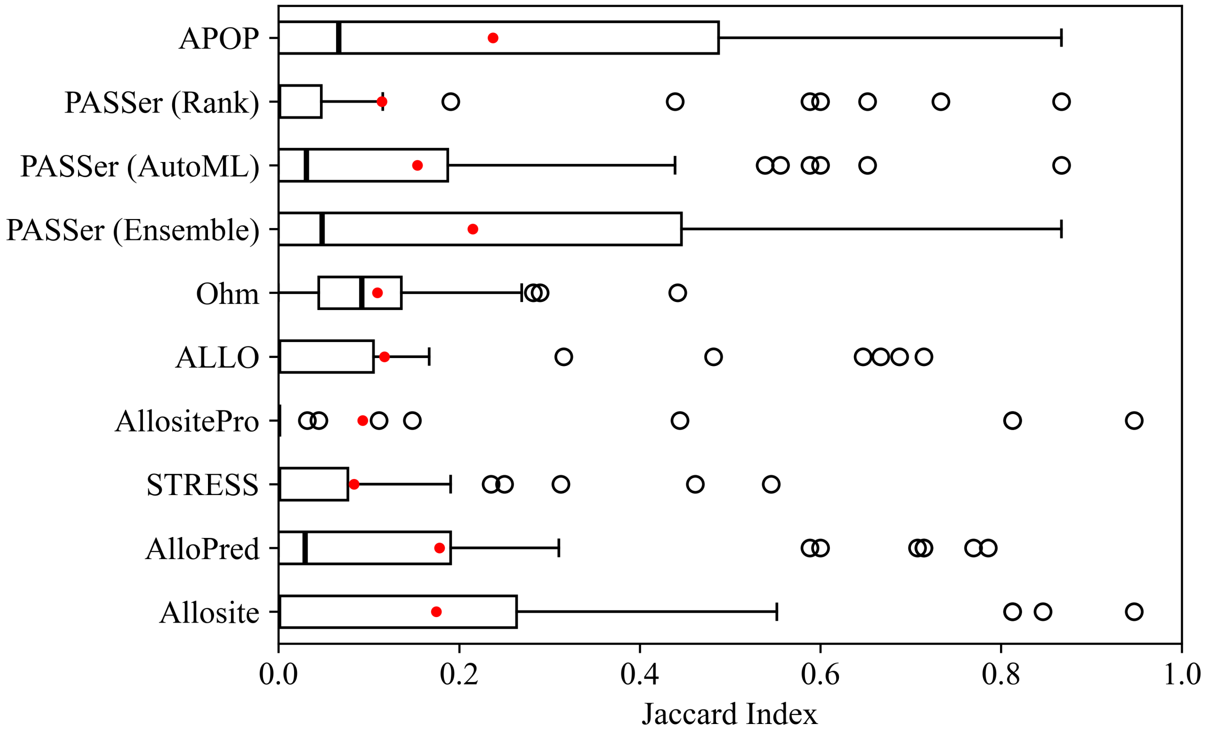

Figure S3. The Jaccard index of each program/web server's topmost predicted allosteric site. This is similar to **Figure 5**, but only monomeric proteins are included (with the multimers excluded). The consistency with **Figure 5** supports that AlloBench is a robust comprehensive benchmarking dataset.

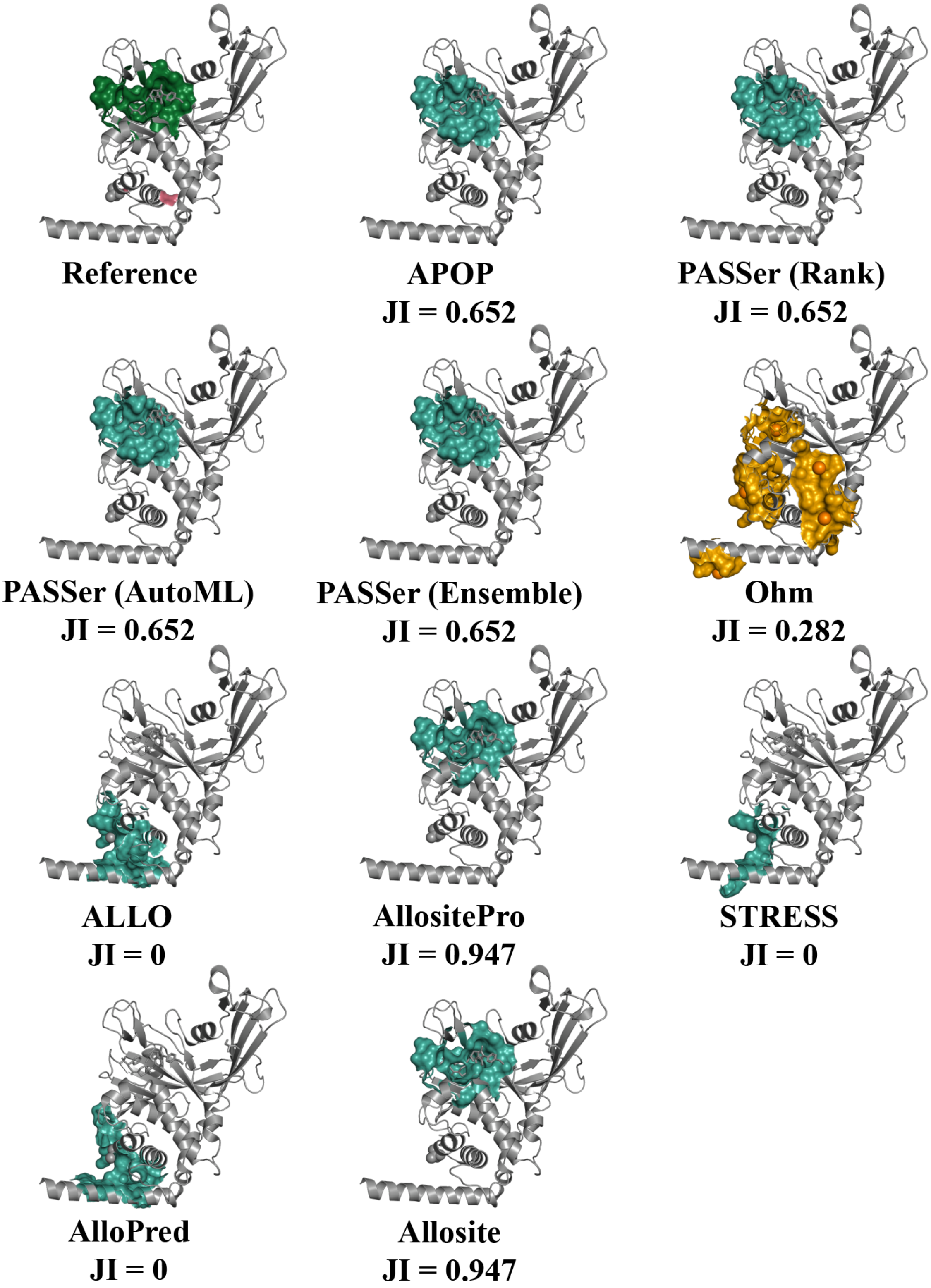

Figure S4: Top-ranked predicted allosteric sites in Human ubiquitin-specific protease 7 (PDB ID: 5N9T) from each web server. The Jaccard index (JI) of each prediction is given below the respective structure. In the reference structure, the allosteric site is colored dark green, and the active sites are colored red. The predicted allosteric sites are colored green. The location of all Ohm-predicted hotspots is shown with orange spheres, and the surface residues within 8 Å are shown in orange. As Ohm does not rank its hotspots, the JI of the best prediction is provided.

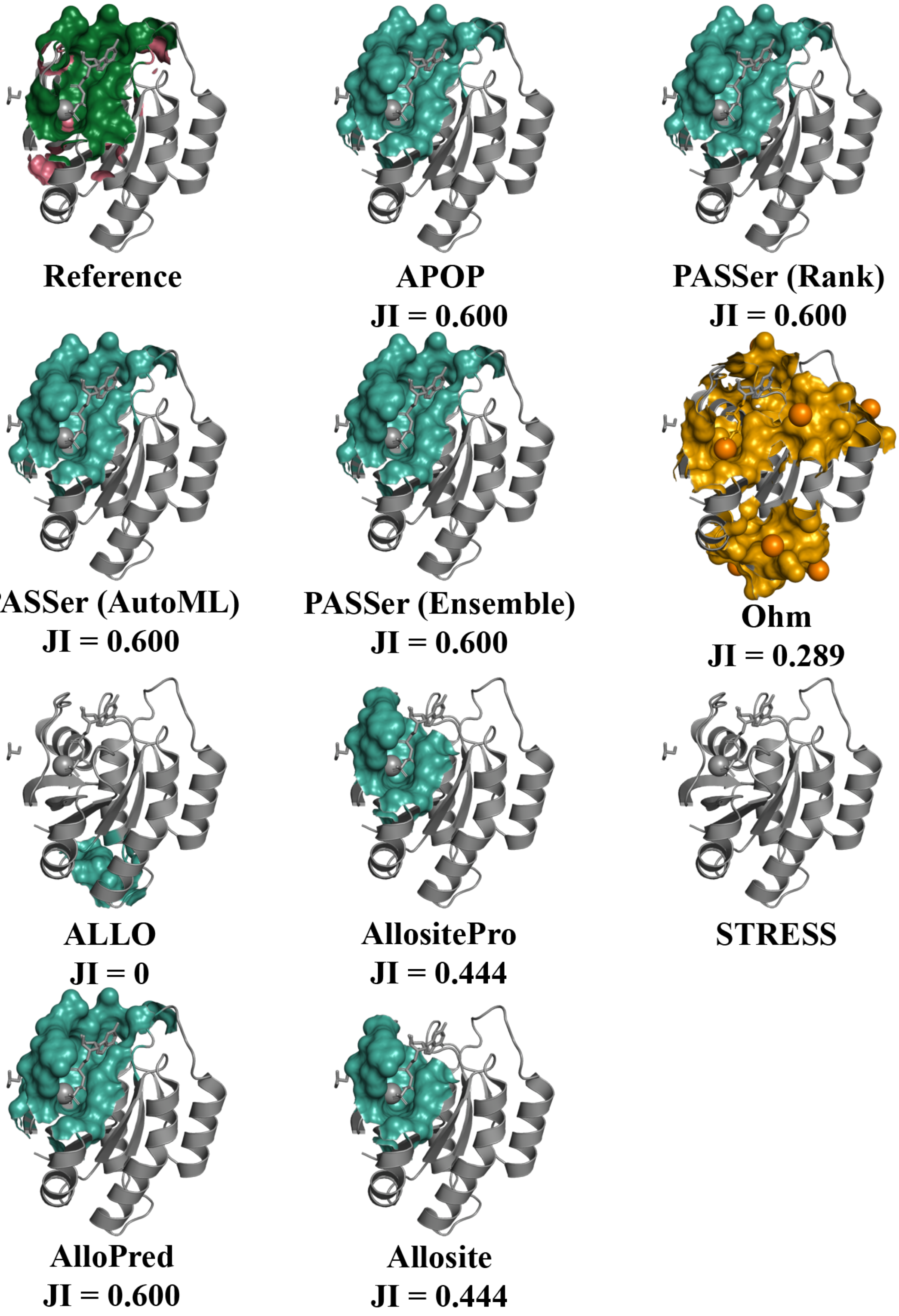

Figure S5: Same as Figure S3, but for the top-ranked predicted allosteric sites in Human GTPases N-Ras (PDB ID: 5UHV) from each web server.

S5. Supporting Tables

Table S1: Essential differences between AlloBench and the previous datasets

**Feature**	**AlloBench** (this work)	**CASBench** [1]	**ASBench** [2]
Allosteric sites	From ASD 2023	From ASD earlier than 2017	From ASD 2014
Active sites	From UniProt + M-CSA	From CSA (earlier version of M-CSA)	None
Synchronization of allosteric and active site residues	Using sequence alignment	Reidentified using ligands	
Allosteric modulator type	All	All	'regular' organic molecules only
Missing residues	Modeled using Promod3		Structures dropped if missing residues were within 4 Å of the allosteric site.
Sampling	None	From CD-HIT sequence alignment clusters	From APoc structural alignment clusters
Quaternary structure	Biological assembly downloaded from PDB	Reconstructed from BIOMT records of PDB files	
Structure resolution	Customizable (default < 4 Å)		< 3 Å

Table S2: Fpocket features used by AlloPred, Allosite, and PASSer.

	**Fpocket Features**	**PASSer**	**Allosite**	**AlloPred**
**1.**	Fpocket Rank			✓
**2.**	Fpocket Score	✓		✓
**3.**	Druggability Score	✓	✓	✓
**4.**	Number of α Spheres	✓	✓	✓
**5.**	Total SASA	✓	✓	✓
**6.**	Polar SASA	✓	✓	✓
**7.**	Apolar SASA	✓	✓	✓
**8.**	Volume	✓	✓	✓
**9.**	Mean local hydrophobic density	✓	✓	✓
**10.**	Mean α sphere radius	✓	✓	✓
**11.**	Mean α sphere solvent accessibility	✓	✓	✓
**12.**	Apolar α sphere proportion	✓		✓
**13.**	Hydrophobicity score	✓		✓
**14.**	Volume score	✓		✓
**15.**	Polarity score	✓	✓	✓
**16.**	Charge score	✓	✓	✓
**17.**	Proportion of polar atoms	✓	✓	✓
**18.**	α sphere density	✓	✓	✓
**19.**	Center of mass - α sphere maximum distance	✓	✓	✓
**20.**	Flexibility	✓	✓	✓
	**Fpocket Derived Features**			
**21.**	Normalized polarity score		✓	
**22.**	Ratio of α sphere density		✓	
**23.**	Ratio of α Spheres		✓	
**24.**	Ratio of apolar α spheres		✓	
**25.**	Ratio of local hydrophobic density		✓	
**26.**	Ratio of maximum distance between the center of mass and all α spheres		✓	

Table S3: Jaccard index of topmost allosteric site prediction from each tool for 100 proteins in the test set.

FAILED: Proteins for which the program encountered an error or could not produce a prediction.

*: A spurious residue returned in the top 3 predictions for the protein from PASSer (Rank), PASSER (AutoML), or PASSER (Ensemble).

**: Proteins for which these AllostiePro and Allosite could not find any allosteric site even after running successfully

Sl No.	PDB ID	APOP	PASSer (Rank)	PASSer (AutoML)	PASSer (Ensemble)	Ohm	ALLO	AllositePro	STRESS	AlloPred	Allosite
1	1A3W	0	0	0.071	0.071	0	0.824	0**	0	0	0
2	1ECB	0.115	0.062	0.115	0.371	0.022	0.393	0**	0	0.115	0.125
3	1LVW	0	0	0	0	0.028	0	0**	0	0	0
4	1MP3	0.737	0.737	0.737	0.737	0	0.105	0.312	0.286	0.750	0.312
5	1NE7	0.007	0.007*	0.007*	0.007*	0.194	0	0.010	0	0.007	0.010
6	1S9I	0.246	0.360	0.246	0.360	0	0.378	0	0.286	0.360	0.194
7	2G88	0	0	0.179	0.179	0	0.481	0	0	0.786	0
8	2J0X	0	0	0	0.400	0	0.032	0**	0	0.400	0
9	2JJX	0.550	0	0.579	0.579	0	0.611	0.208	0	0	FAILED
10	2OZ6	0.056	0.739	0.739	0.056	0.400	0	0**	0.120	0.056	0.333
11	2PTM	0	0.722	0.722	0.722	0	0.65	0.684	0	FAILED	0
12	3EK5	0.138	0.138	0.138	0	0	0.141	0.069	0.143	0.138	0.069
13	3HQP	0	0	0	0	0	0	0	0	0	0
14	3HQQ	0	0	0	0	0	0	0	0	0	0
15	3JAF	0.045	0.045	0.049	0.045	0	0.205	0	0	0.045	0
16	3PUO	0	0.211	0.381	0.150	0	0.079	0.209	0.056	0.381	0.209

17	3TCE	0.184	0.184	0.184	0.184	0	0.078	0**	0.188	0.184	0.106
18	3V4F	0	0	0	0*	0	0.091	0	0	0	0
19	3WGG	0	0.600	0	0.600	0.029	0.139	0.012	0.031	0.600	0.012
20	3X1M	0.537	0.550	0.550	0.550	0	0.421	0.393	0.280	0.537	0.393
21	4C8B	0.313	0	0.313	0.065	0.044	0.020	0.303	0	0.292	0.303
22	4COJ	0	0	0.125	0.586	0	0.024	0	0	0	0.515
23	4DLR	0	0*	0*	0*	0	0	0	FAILED	0	0
24	4DLV	0	0	0	0	0	0	0	FAILED	0	0
25	4DLX	0	0	0*	0*	0	0	0	FAILED	0	0
26	4DLY	0.115	0.115	0.115	0.115	0	0	0	FAILED	0.115	0.100
27	4DLZ	0.074	0.074*	0.074*	0.074*	0	0	0.148	FAILED	0.074	0.125
28	4HYV	0	0	0	0	0	0	0	0	0	0
29	4MHD	0	0.111*	0.105*	0.526	0.026	0.027	0**	0.111	0	0.017
30	4MI4	0	0.030*	0.030*	0.030*	0	0.164	0**	0.235	0	0**
31	4NES	0.317	0	0.317	0.317	0	0	0**	0	0.317	0.345
32	4OV9	0.343	0.033	0.343	0.343	0	0.080	0.273	0.294	0.343	0.149
33	4P02	0	0	0	0	0	0	0	0	0	0.015
34	4PA0	0	0	0	0	0	0	0**	0	0.714	0
35	4PFK	0	0	0	0	0	0.765	FAILED	0.042	0	FAILED
36	4RER	0	0	0	0	0	0	0	0	0	0
37	4RUK	0.600	0.426	0.426	0.426	0	0.062	0.486	0.069	0.426	0.486

38	4UC5	0.036	0	0.026	0.026	0	0	0.018	0	0.036	0.018
39	4YPL	0	0.024	0.024	0.024	0	0	0.181	0.091	0.023	0
40	4YW8	0.286	0	0.310	0.286	0	0.132	0**	0.462	0.310	0.360
41	4ZJI	0.439	0.439	0.439	0.439	0	0	0	0.077	FAILED	0.097
42	4ZSG	0.867	0.867*	0.867	0.867	0	0.647	0.812	0.043	0.056	0.812
43	4ZSJ	0.824	0*	0.065*	0.706	0	0.688	0.812	0	0.061	0.812
44	4ZSL	0.733	0.733	0.077	0.733	0	0.714	0**	0.235	0.065	0.846
45	5AA4	0.029	0	0.029	0.707	0	0	0.032	0	0.707	0
46	5DL1	0.260	0.260	0.260	0.247	0	0.247	FAILED	0	0.247	FAILED
47	5DR2	0.043	0	0.043	0.043	0.032	0	0.044	0.250	0.043	0.044
48	5F6W	0.059	0*	0*	0*	0	0.037	FAILED	FAILED	0	0
49	5F6Y	0	0*	0.556*	0.556*	0	0	0	FAILED	0	0
50	5FTJ	0	0	0	0.459	0	0	0**	0	0	0.003
51	5GRF	0	0	0	0	0	0	0**	0.158	0	0
52	5H08	0.600	0	0	0	0	0.045	0	0	FAILED	0.262
53	5H3Q	0.538	0	0.538	0.538	0.053	0.105	0	0.069	FAILED	0.396
54	5HUE	0	0	0.600	0.600	0	0.600	0.083	0.095	0	0**
55	5IKP	0	0	0	0	0	0.156	FAILED	0	0	0
56	5IM3	0	0	0	0	0.079	0	0**	0	0	0
57	5IN4	0.160	0	0	0.160	0	0.065	FAILED	0.087	0	0.256
58	5IXE	0.057	0.833	0.132	0.132	0	0	0**	0	0.057	0.278

59	5J6F	0.069	0	0	0.069	0	0	0**	0	0	0**
60	5KDE	0	0	0.267	0	0	0.235	0**	0	0	0**
61	5KDF	0	0	0.312	0.312	0	0.25	0**	0	0	0.208
62	5KWI	0.700	0*	0*	0*	0	0	0.111	0.062	0	0.111
63	5KWJ	0.048	0*	0.05	0*	0	0.093	0**	0.545	0	0**
64	5LVX	0.109	0.286*	0.194*	0*	0	0	0.078	0	0	0.079
65	5M0E	0.381	0	0.381	0.381	0	0.316	0**	0	0	0.333
66	5N9T	0.652	0.652	0.652	0.652	0	0	0.947	0	0	0.947
67	5NGZ	0	0	0	0	0.083	0.667	0	FAILED	0.769	0
68	5NN4	0	0	0	0	0.050	0	0**	0	0	0
69	5NQB	0.137	0	0.118	0.118	0.038	0.083	0	0	0.137	0
70	5NQQ	0	0	0*	0*	0	0	0	0	0	0
71	5NZK	0.077	0	0	0.077	0	0	0	0	0	0
72	5NZM	0.053	0.053	0.053	0.053	0	0	0	0	0.053	0
73	5OFU	0	0	0	0	0	0.464	0**	0.211	0	0.684
74	5OLK	0.115	0	0	0	0	0	0**	0.111	0	0
75	5T45	0	0.588	0.588	0	0	0	0**	0	0.588	0**
76	5TF9	0.448	0	0	0.448	0	0	0	0	FAILED	0.264
77	5TKV	0.144	0.144	0.144	0.144	0	0	0**	0	0.144	0.100
78	5U1U	0.088	0.053	0.088	0.088	0	0.444	0.045	0	0.088	0.462
79	5U1V	0	0.048	0.048	0	0.054	0	0**	0.043	0.130	0.441

80	5U1W	0	0	0	0	0	0.088	0.348	0.250	0.087	0.450
81	5U1X	0.762	0.037	0.762	0.762	0	0	0.696	0	0.143	0.500
82	5U1Y	0	0.042	0	0	0	0.684	0**	0.042	0	0.500
83	5UGH	0.583	0	0	0.583	0.071	0	0	0	0	0
84	5UHV	0.600	0.600	0.600	0.600	0	0	0.444	FAILED	0.600	0.444
85	5URJ	0.200	0	0	0	0	0	0**	0	0	0
86	5UXM	0	0*	0*	0*	0	0.474	0	0	0	0.239
87	5VDH	0	0	0	0.023	0	0.361	FAILED	0	0.016	0
88	5VDI	0	0	0.353	0.400	0	0.353	FAILED	0	0	FAILED
89	5VYJ	0	0.562	0	0	0	0	0**	0	0	0.008
90	5WMV	0.190	0.190	0.190	0.190	0	0.105	0**	0	0.19	0.123
91	5X9U	0.122	0	0.122	0	0	0.064		0	0.227	0.304
92	5XZR	0.500	0	0	0.500	0	0.073	0	0.190	0	0
93	5Y2F	0.063	0.065	0.065	0.065	0.136	0	0**	0	0.063	0**
94	5Y66	0.029	0.032	0.032	0.032	0	0.167	0	0	0.029	0.552
95	5YVE	0	0	0	0	0	0	0**	0	0	0.517
96	6B21	0	0	0	0	0.103	0	0**	0	0	0
97	6CGN	0.538	0	0	0	0	0	0	0	0	0
98	6D7O	0.220	0	0.220	0.220	0	0.278	0**	0	0	0
99	6H8R	0	0	0	0	0	0	0	0.312	FAILED	0
100	6H8S	0	0	0*	0	0	0.027	0	0.176	0.714	0

Table S4: Distribution of AlloBench proteins according to source organism.

Organism	Count	Percentage
Human	862	42.38%
Bacteria	450	22.12%
Viruses	321	15.78%
Rat	92	4.52%
Rabbit	73	3.59%
Pig	47	2.31%
Archaea	36	1.77%
Mouse	35	1.72%
Yeast	24	1.18%
Bovine	17	0.84%
Chicken	15	0.74%
Others	62	3.05%

Table S5: Distribution of AlloBench proteins according to function.

Target Protein Function	Count	Percentage
Transferase	595	29.25%
Hydrolase	477	23.45%
Transport	284	13.96%
Other Proteins	148	7.28%
Oxidoreductase	130	6.39%
Lyase	112	5.51%
Transcription Factor	89	4.38%
Receptor	75	3.69%
Motor Protein	45	2.21%
Ligase	33	1.62%
Isomerase	17	0.84%
GPCR	16	0.79%
Chaperone	10	0.49%
Initiation Factor	3	0.15%

Table S6: Distribution of AlloBench entries by the type of allosteric modulator

Type of Allosteric Modulator	Count	Percentage
Lig	1931	90.19%
Ion	164	7.66%
Pep	41	1.91%
Lig + Ion	4	0.19%
Gas	1	0.05%

Table S7: Distribution of AlloBench proteins according to oligomeric state.

Oligomeric State	Count	Percentage
Monomer	618	30.38%
Homo 2-mer	597	29.35%
Homo 4-mer	363	17.85%
Hetero 4-mer	137	6.74%
Hetero 2-mer	109	5.36%
Homo 6-mer	50	2.46%
Hetero 3-mer	32	1.57%
Homo 3-mer	32	1.57%
Hetero 12-mer	25	1.23%
Homo 8-mer	21	1.03%
Hetero 6-mer	17	0.84%
Hetero 8-mer	17	0.84%
Homo 5-mer	5	0.25%
Hetero 28-mer	3	0.15%
Homo 12-mer	2	0.10%
Hetero 5-mer	2	0.10%
Hetero 21-mer	2	0.10%
Hetero 7-mer	1	0.05%
Homo 14-mer	1	0.05%

Table S8: Percentage of proteins with Jaccard Index (JI) above the cutoff in each column for the topmost allosteric site prediction from each program for 100 proteins in the test set. Ohm does not rank its predicted allosteric sites; only the first prediction is considered here.

Program	JI > 0	JI > 0.1	JI > 0.2	JI > 0.3	JI > 0.4	JI > 0.5
**APOP**	54	38%	26%	22%	18%	15%
**PASSer (Rank)**	38%	24%	18%	15%	14%	12%
**PASSer (AutoML)**	57%	40%	27%	23%	15%	13%
**PASSer (Ensemble)**	60%	43%	34%	31%	23%	18%
**Ohm**	17%	4%	1%	1%	0%	0%
**ALLO**	52%	34%	25%	20%	15%	10%
**AllositePro**	26%	17%	14%	11%	7%	5%
**STRESS**	33%	20%	12%	3%	2%	1%
**AlloPred**	46%	29%	20%	17%	11%	10%
**Allosite**	51%	38%	31%	24%	15%	8%
